# Computer simulations explain the anomalous temperature optimum in a cold-adapted enzyme

**DOI:** 10.1038/s41467-020-16341-2

**Published:** 2020-05-26

**Authors:** Jaka Sočan, Miha Purg, Johan Åqvist

**Affiliations:** Department of Cell & Molecular Biology, Uppsala University, Biomedical Center, Box 596, SE-751 24, Uppsala, Sweden

**Keywords:** Enzymes, Theoretical chemistry

## Abstract

Cold-adapted enzymes from psychrophilic species show the general characteristics of being more heat labile, and having a different balance between enthalpic and entropic contributions to free energy barrier of the catalyzed reaction compared to mesophilic orthologs. Among cold-adapted enzymes, there are also examples that show an enigmatic inactivation at higher temperatures before unfolding of the protein occurs. Here, we analyze these phenomena by extensive computer simulations of the catalytic reactions of psychrophilic and mesophilic α-amylases. The calculations yield temperature dependent reaction rates in good agreement with experiment, and also elicit the anomalous rate optimum for the cold-adapted enzyme, which occurs about 15 °C below the melting point. This result allows us to examine the structural basis of thermal inactivation, which turns out to be caused by breaking of a specific enzyme-substrate interaction. This type of behaviour is also likely to be relevant for other enzymes displaying such anomalous temperature optima.

## Abstract (web summary)

Enzymes from organisms inhabiting cold environments (psychrophiles) have adapted to catalyzing chemical reactions at near freezing temperatures. Here – using molecular dynamics simulations – the authors analyze cold adaptation of psychrophilic α-amylase and provide the structural basis for its low anomalous temperature optimum: the increased mobility of a surface loop involved in substrate interaction.

## Introduction

Among the different types of extreme environments found on Earth, cold areas with permanent temperatures below 5 °C cover the major part of the planet, particularly due to the large oceans. Such a cold environment would be detrimental for biochemical processes in species that have the same internal temperature as their surroundings, had evolution not adapted them to this situation. A fundamental question here is how the enzymes of such psychrophilic organisms can overcome the exponential retardation of chemical reaction rates at low temperatures. That is, most mesophilic enzymes lose a large fraction of their maximal activity near the freezing point of water, while cold-adapted enzymes have managed to maintain reaction rates high enough to sustain life in the cold. There is now ample evidence from kinetic studies of numerous orthologous mesophilic–psychrophilic enzyme pairs that cold-adapted enzymes have shifted their thermodynamic activation parameters so that the activation enthalpy (Δ*H*^‡^) is reduced, which is partly counterbalanced by an increased activation entropy penalty (Δ*S*^‡^ is more negative)^1–4^. This seemingly universal characteristic has the effect that the exponential dampening of rates at lower temperatures is less pronounced, since the reaction rate $k_{{\mathrm{rxn}}} = C \cdot T{\mathrm{e}}^{ - \Delta G^\ddagger /RT} = C \cdot T{\mathrm{e}}^{ - \Delta H^\ddagger /RT}{\mathrm{e}}^{\Delta S^\ddagger /R}$ according to standard transition-state theory (where $\Delta G^\ddagger = \Delta H^\ddagger - T\Delta S^\ddagger$ is the activation free energy). It is thus now generally accepted that the main adaptive feature of psychrophilic enzymes is the redistribution of the thermodynamic activation parameters compared with mesophilic and thermophilic orthologs, while the activation free energies are usually similar at room temperature^1–5^.

While the shifted activation enthalpy–entropy balance is what is directly responsible for the higher rates at low temperature, a lower melting temperature is another characteristic of cold-adapted enzymes. Hence, compared with mesophilic enzymes, one typically finds that *T*_m_ is shifted downward by about 5–20 °C^2,3^. It is usually the opposing effects of the exponential rate increase at higher temperatures, and the eventual melting of the enzyme that gives rise to a temperature optimum (*T*_opt_) of the catalytic rate. It should be noted here that there is another important difference between psychrophilic enzymes and mesophilic/thermophilic ones, in that the former generally work far away from *T*_opt_, while the latter operate closer to the optimum and the melting temperature. This means that the evolutionary pressure on protein stability at the physiological working temperature is bound to be considerably weaker for psychrophilic enzymes, which may be one reason for why their *T*_m_ has drifted toward lower values compared with mesophilic orthologs^5,6^.

The structural origins of both the change in thermodynamic activation parameters and the lower melting temperature have been linked to a higher protein flexibility of cold-adapted enzymes^2,4^, especially in surface loop regions^4,5,7–12^. In particular, computer simulations that directly evaluated free energy barriers for the catalyzed reactions^9–13^ have shown that the mobility of active-site residues is generally very similar in psychrophilic and mesophilic enzyme orthologs, while surface loop mobilities may differ considerably and strongly affect the balance between Δ*H*^‡^ and Δ*S*^‡^. This finding is entirely in line with the fact that multiple sequence alignments between orthologous psychrophilic and mesophilic enzymes reveal that characteristic mutations are typically located in such loop regions^9–11^.

**Fig. 1:**
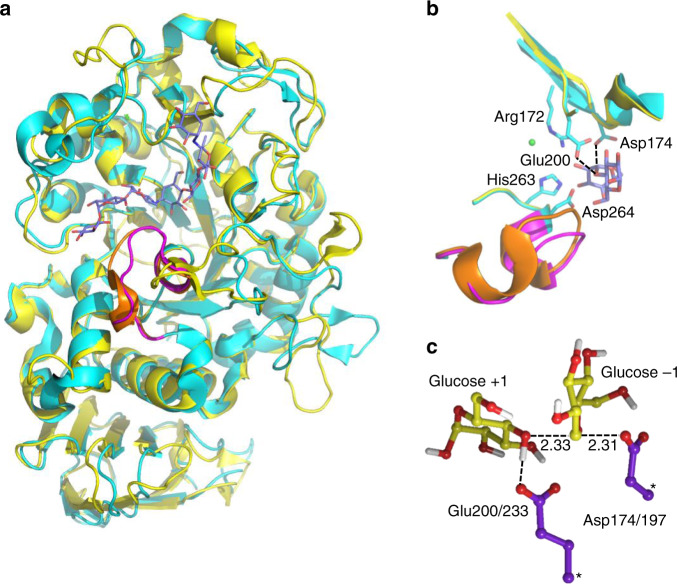
Structures of the psychrophilic and mesophilic α-amylases and their active site. **a** View of the two enzymes (AHA in cyan and PPA in yellow)^36,38^ with the bound substrate (blue carbons). The L7 loop partly covering the substrate is shown in purple for AHA and orange for PPA. **b** Close-up view of the active site with sidechains shown for AHA and the interactions between the nucleophile (Asp174) and general acid (Glu200) indicated with dashed lines (a crystallographically observed Cl^−^ ion is shown in green). **c** Optimized transition-state structure from DFT calculations of the model reaction in solution (atoms marked with asterisks were fixed).

The most well-studied psychrophilic enzyme is probably the α-amylase from the Antarctic bacterium *Pseudoalteromonas haloplanktis* (AHA), largely due to the efforts of Gerday, Feller, and coworkers^3,14–17^. This multidomain enzyme (Fig. 1) has thus served as a model for cold adaptation, and it has been shown to be a much faster catalyst at low temperatures than its mesophilic and thermophilic orthologs from pig pancreas (PPA) and *Bacillus amyloliquefaciens*, respectively^14^. It is also found to be more heat labile with a melting temperature that is ~15 °C lower than the porcine pancreatic variant. Furthermore, it was suggested that the decreased stability of AHA originates from localized increases in flexibility (or local unfolding) of structural regions close to the active site, which may be connected to the higher *K*_m_ values observed for similar substrates, compared with those in PPA^17^. It has also been shown that introduction of mutations into AHA, encompassing structural features typical for PPA, diminishes the psychrophilic properties of the former^15^. In addition, a few computational studies have addressed the catalytic mechanism of α-amylases with QM/MM simulations^18,19^, which corroborate the standard lysozyme-like mechanism^20^. Hence, the glycosylation step is predicted to be rate-limiting, where a carboxylate moiety of the enzyme (Asp174 in AHA) attacks the glycosidic bond to yield a covalent enzyme–substrate intermediate, concomitantly with leaving group protonation by a carboxylic acid moiety (Glu200 in AHA). Classical MD simulations comparing AHA and PPA and focusing on the dynamic properties of the apo-enzymes have also been reported^21,22^.

A particularly interesting feature of the cold-adapted α-amylase is that its rate optimum at 25–30 °C is well below the melting temperature of 44 °C, indicating that inactivation of AHA is due to something other than global unfolding. This is not the case for the mesophilic and thermophilic orthologs where the optimum essentially coincides with *T*_m_^14^. While a coincidence of optimum rate and the onset of unfolding is also found for most psychrophilic enzymes^23–27^, there are other such examples where the optimum occurs significantly earlier than melting of the protein^28,29^. This has usually been interpreted in terms of local unfolding of the enzyme or an unstable active site^14,28,29^, but a more exotic hypothesis posits that there is a non-negligible heat capacity difference between the transition and ground state ($\Delta C_P^\ddagger$)^30–32^. This would give rise to a strong temperature dependence of Δ*H*^‡^ and Δ*S*^‡^, leading to curved free energy and activity plots.

It is important here to emphasize that in order to computationally address the outstanding questions of how psychrophilic enzymes can alter the activation enthalpy–entropy balance, and how catalytic rate optima could arise far from the melting temperature, it is absolutely necessary to be able to directly calculate the temperature dependence of the free energy barriers and rates^5^. Thus, to establish the origins of these effects in AHA, we constructed an empirical valence bond (EVB) model^33,34^ for the rate-limiting glycosylation step in the α-amylases, based on density functional theory (DFT) quantum mechanical calculations. With this EVB model, we are able to carry out very extensive molecular dynamics (MD) free energy calculations over a wide temperature range, and uncover the origins of these interesting catalytic properties of the psychophilic α-amylase.

## Results

### EVB and DFT modeling of the reaction of α-amylases

The predominant enzymatic activity of α-amylases is the hydrolysis of α-1,4 bonds within starch molecules. The catalytic reaction first involves a glycosylation step resulting in covalent enzyme–substrate intermediate, which is subsequently hydrolyzed by a water molecule in a deglycosylation step, thereby restoring the enzyme-active site^35,36^. The glycosylation step has been shown to be rate-limiting for α-amylases^37^, and was described here by the EVB method^33,34^, using a five-residue glucose oligomer as the substrate in complex with both the psychrophilic *P. haloplanktis* α-amylase (AHA)^36^ and the mesophilic porcine pancreatic α-amylase (PPA)^38^. To calibrate the EVB model, we employed DFT calculations with a continuum solvent model for a small reference system consisting of two carboxylic sidechains, representing the nucleophile and general acid of the reaction (Asp174/197 and Glu200/233 in AHA/PPA, respectively), set to cleave the linkage between two glucose residues. Here, the geometry of reacting moieties represents that found in typical α-amylase active sites (see “Methods”).

**Fig. 2:**
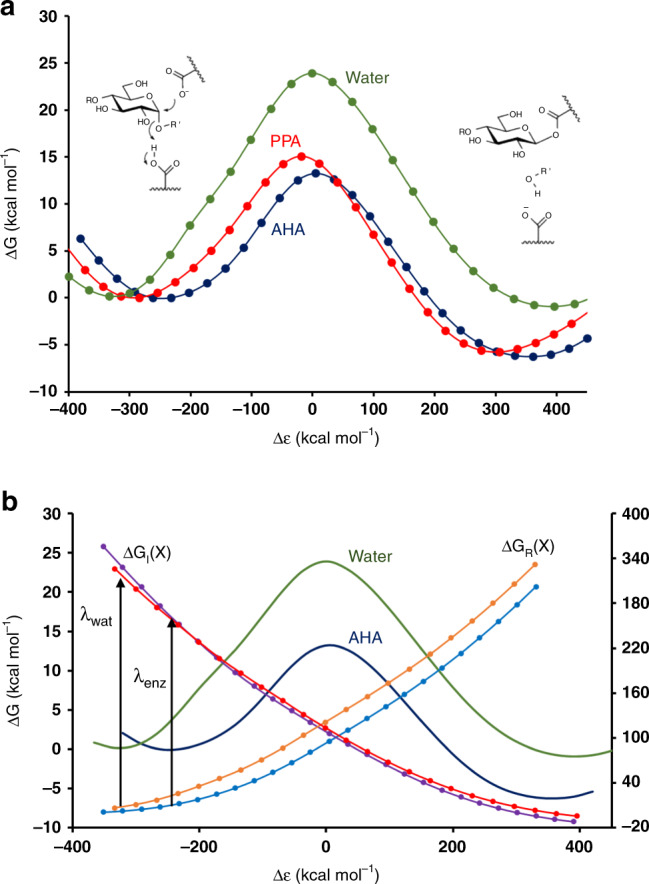
Reaction free energy profiles. **a** Calculated free energy profiles from reactants to the covalent intermediate for the reference reaction in solution (green) and two α-amylases: psychrophilic AHA (blue) and mesophilic PPA (green). Δ*ε* is the generalized reaction coordinate^33,34^, and the s.e.m. for the free energy barriers is 0.08–0.14 kcal mol^−1^ (from ~300 replicate simulations). **b** Illustration of the reorganization energy effect in AHA. The reaction free energy profiles for the reference reaction (green) and AHA (blue) are shown (left-hand *y* axis) together with the diabatic free energy functions corresponding to the pure reactant (Δ*G*_R_— orange and light blue for water and enzyme, respectively) and intermediate (Δ*G*_I_— red and purple for water and enzyme, respectively) states in the two cases (right-hand *y* axis). The reorganization energies are indicated with vertical arrows.

The reaction energetics in water for this reference system was obtained by M06-2X/6-311 + G(2d,2p) DFT calculations with the SMD solvent model^39,40^. Geometry optimization and transition-state (TS) search yielded a concerted oxocarbenium-like transition state with a sole imaginary frequency, where the general acid is essentially deprotonated, and the anomeric carbon distances to the nucleophile and leaving oxygen are 2.31 and 2.33 Å, respectively (Fig. 1c). This type of TS is comparable with that found in several studies reported earlier^18,20^. The activation and reaction free energy at 25 °C for the glycosylation reaction in water were thus calculated as $\Delta G_{{\mathrm{ref}}}^\ddagger = 23.9$ and $\Delta G_{{\mathrm{ref}}}^0 = - 1.1$ kcal mol^−1^, respectively (Supplementary Data 1), notably with a free energy barrier that is about 10 kcal mol^−1^ higher than that observed for the corresponding enzyme reaction^15,16^. These results were used to parameterize an (uncatalyzed) EVB reference free energy surface in water, which can then be used to analyze the catalytic effect of the rest of the enzyme, apart from the groups directly involved in the chemistry. Hence, the resulting EVB model exactly reproduces the energetics of the solution reaction, as obtained by the DFT calculations (Fig. 2). The effect of the surrounding enzyme is then obtained by carrying out EVB/MD free energy simulations of the same reaction in the enzyme-active site, where the entire protein is solvated in water. As noted above, it is the efficiency of the EVB representation of the reaction surface that allows for extensive sampling by MD and evaluation of thermodynamic activation parameters. It should also be noted here that the general acid corresponding to Glu200/233 must be treated as protonated in the reactant state since experimental pH-rate profiles unambiguously show that this is the case for the enzyme–substrate complex^41,42^.

### Origin of the catalytic effect in the α-amylases

The results of the EVB/MD simulations in the psychrophilic (AHA) and mesophilic (PPA) α-amylases are summarized in Fig. 2 in terms of average reaction free energy profiles, each from ~300 independent simulations at 25 °C. It can immediately be seen that the two enzymes lower the activation barrier by ~10 kcal mol^−1^ compared with the reference reaction in water. Moreover, the psychrophilic enzyme is predicted to be faster than the mesophilic at room temperature, and the calculated free energy barriers are 13.3 ± 0.08 and 15.1 ± 0.14 kcal mol^−1^ for AHA and PPA, respectively (error bars ± 1 s.e.m. from ~300 replicate simulations). These results agree very well with the available experimental data, which imply free energy barriers of ~14 kcal mol^−1^ for typical short substrates and an approximately threefold higher *k*_cat_ value for AHA compared with PPA^14,16^. The first question that arises from our calculations is where the 10 kcal mol^−1^ catalytic effect of the α-amylases, compared with the solution reaction, comes from. Actually, a survey of the literature regarding computational modeling of glucosidases indicates that the question of what causes their high catalytic rates basically remains unanswered. That is, computational studies in the field have mainly been focused on details of the catalytic mechanisms (inverting, retaining, concerted, stepwise etc.) and on the resulting free energy barriers, without comparison with any uncatalyzed reference reaction^18–20,43^. A notable exception is the pioneering work of Warshel on lysozyme^33,44^, who ascribed the catalytic effect mainly to electrostatic stabilization of a proposed ionic oxocarbenium intermediate by the negatively charged nucleophilic carboxylate group. However, such a mechanism can now be ruled out as the covalent enzyme-bound intermediate has been observed experimentally^45,46^, and reliable QM/MM calculations have provided convincing results in support of the covalent reaction pathway^20^.

As virtually all computational studies of glucosidase reactions have employed QM/MM methods, where reference reactions in solution are generally avoided, there have been no uncatalyzed reactions to compare with. However, in our case, the results clearly show that just putting the nucleophilic group, general acid, and substrate in the same geometry as in the α-amylases, but surrounded by water, does not at all suffice for achieving the low activation barriers found in the enzymes. It is also clear that while part of the 10 kcal mol^−1^ barrier reduction originates from stabilization of the covalent intermediate, this does not explain the entire effect (Fig. 2a; Supplementary Fig. 1). Here, analysis of the calculated energetics shows that it is primarily the reorganization free energy^33,34,47^ that has been significantly reduced in the enzymes compared with the solution reaction (Fig. 2b). This type of transition-state stabilization is one of the major features of enzyme-catalyzed reactions and reflects the preorganization of the active site^48^, which diminishes the energetic cost of reorienting polar groups surrounding the substrate as its charge distribution changes along the reaction path^47–49^. Such (solvent) reorganization is otherwise a major contributor to the free energy barrier of uncatalyzed reactions in water. The magnitude of the reorganization energy is also directly reflected by the energy gap between the reactant and product states at the reactant minimum. As can be seen from Fig. 2, the reactant minimum in the enzymes is shifted toward smaller absolute values of this energy gap than in the reference reaction, demonstrating that the required reorganization between reactant and products (covalent intermediate) is significantly smaller in the enzymes.

As the negative charge on Asp174/197 in the reactant state ends up on Glu200/233 in the intermediate (Fig. 2a), this migration (delocalization) of charge requires a substantial reorientation of water molecules in the solution reaction, with a large associated free energy cost. In the enzymes, on the other hand, the two carboxylates are largely shielded from solvent, and their negative charge is instead stabilized by interactions with polar groups of the protein and substrate. In particular, the sidechain of Arg172/195 is located between Asp174/197 and Glu200/233, and can thus form ionic interactions with the charged carboxylate, both in the reactant and intermediate states, with only a small reorganizational cost (Fig. 1b).

### Temperature dependence of the AHA and PPA reactions

**Fig. 3:**
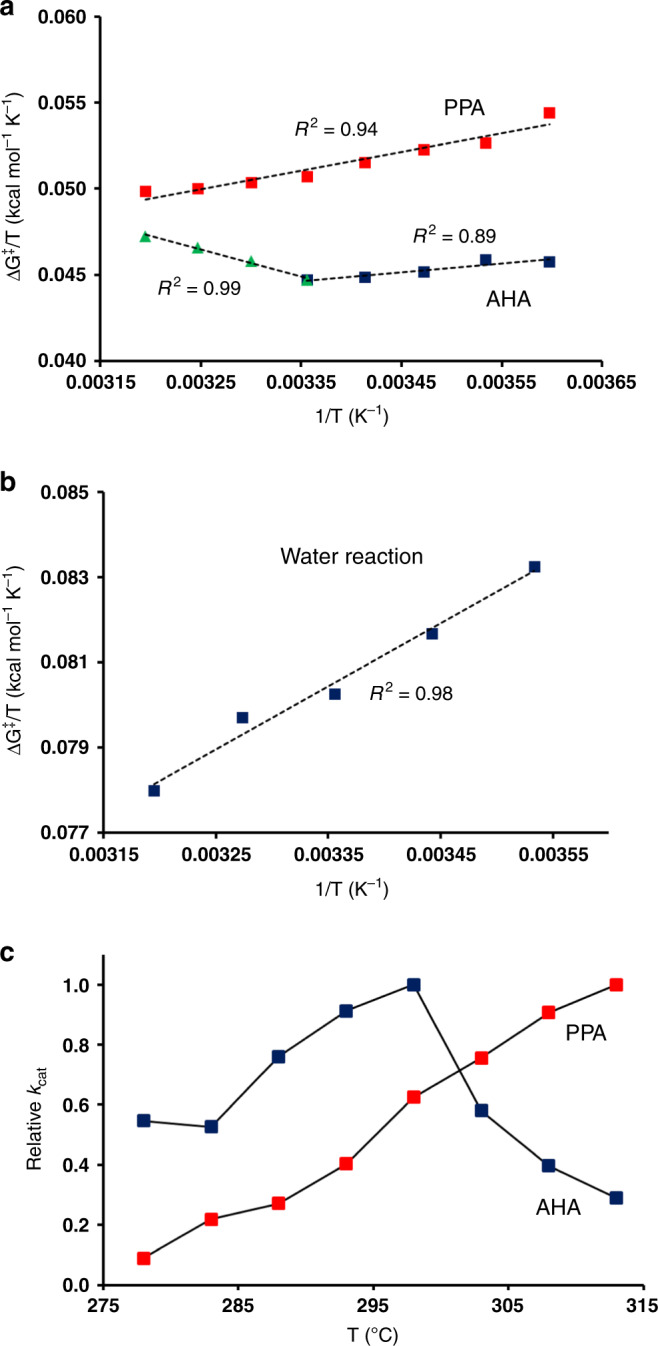
Temperature dependence of the glycosylation reaction. Arrhenius plots of Δ*G*^‡^/*T* vs. 1/*T* for the catalyzed reactions in (**a**) AHA and PPA and (**b**) for the reference reaction in water. Linear regression fits from which thermodynamic parameters are calculated are denoted by dashed lines. **c** Calculated temperature dependence of the relative rate constants *k*_cat_ for AHA (blue) and PPA (red). The s.e.m. for the calculated free energy barriers is 0.08–0.14 kcal mol^−1^ (from ~300 replicate simulations).

We calculated the temperature dependence of the glycosylation step in both AHA and PPA by performing ~300 independent EVB/MD calculations of free energy profiles for each enzyme at eight different temperatures between 5 and 40 °C. From these simulations, Arrhenius plots of Δ*G*^‡^/T vs. 1/*T* were constructed in order to obtain the thermodynamic components of the free energy barrier (Fig. 3). It can immediately be seen from the Arrhenius plots that while PPA essentially shows a linear relationship over the entire temperature range (*R*^2^ = 0.94), there is evidently a break in the plot for AHA. For PPA, the linear relation gives values of Δ*H*^‡^ = 10.8 and *T*Δ*S*^‡^ = −4.3 kcal mol^−1^ at 15 °C. In the case of AHA, the corresponding values are Δ*H*^‡^ = 5.2 and *T*Δ*S*^‡^ = −7.8 kcal mol^−1^, if the entire lower temperature range 5–25 °C is considered, and Δ*H*^‡^ = 6.5 and *T*Δ*S*^‡^ = −6.6 kcal mol^−1^ if one restricts the range to 10–25 °C (as done experimentally for AHA^16^). The predicted values above agree reasonably well with those reported from experiments, where Δ*H*^‡^ = 11.1 and *T*Δ*S*^‡^ = −2.9 kcal mol^−1^ for PPA and Δ*H*^‡^ = 8.3 and *T*Δ*S*^‡^ = −5.1 kcal mol^−1^ for AHA at 15 °C^16^. Moreover, it is clear that the computer simulations capture the shift of the activation enthalpy–entropy balance that is observed experimentally both for this and other orthologous mesophilic–psychrophilic enzyme pairs. That is, AHA shows a smaller Δ*H*^‡^ and a more negative *T*Δ*S*^‡^ than PPA. For comparison, we also calculated the corresponding Arrhenius plot for the uncatalyzed reference reaction, which yields Δ*H*^‡^ = 14.8 and *T*Δ*S*^‡^ = −8.9 kcal mol^−1^ at 15 °C (Fig. 3b). This shows that the two enzymes are able to reduce both Δ*H*^‡^ and −*T*Δ*S*^‡^, but that the faster psychrophilic enzyme has a larger effect on the enthalpy term. Hence, the transition-state stabilization evidently has both enthalpic and entropic contributions.

It is noteworthy that the break in the Arrhenius plot for AHA occurs precisely where the elusive temperature optimum is observed for the psychrophilic α-amylase (~25 °C) which, as noted above, does not reflect thermal unfolding of the enzyme^14^. Hence, if we convert our free energy barriers to rate constants (*k*_cat_), it can be seen that the calculations predict a rate optimum that coincides with that observed in kinetic experiments (Fig. 3c). This is the first time that such a nontrivial reaction rate behavior has been seen from computer simulations, and this unexpected finding now allows us to explore the cause of the anomalous thermal inactivation in a well-defined enzyme case.

### Origin of the thermal inactivation of psychrophilic α-amylase

**Fig. 4:**
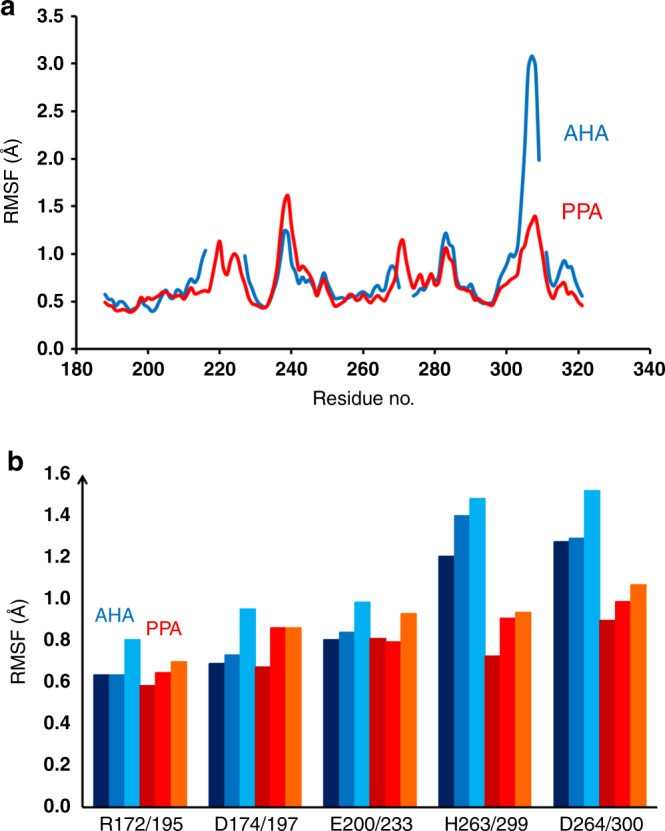
Mobility differences between the psychrophilic and mesophilic enzymes. **a** Calculated average backbone positional root-mean-square fluctuations per residue in the reactant state for AHA (blue) and PPA (red) in the sequence region comprising the active site and L7 loop (PPA residue numbering is used due to sequence insertions). **b** Average sidechain RMSF for active-site residues at 10, 25, and 40 °C. AHA results are shown in dark, medium, and light blue with increasing temperature and PPA results in a corresponding red scale.

It is immediately clear from the present EVB/MD simulations that the cause of the rate decline for AHA above 25 °C does not reflect unfolding of the protein. This is as expected since the timescale of the simulations (sub-μs) would not be able to capture any global melting transition. In order to monitor possible temperature-dependent structural changes near the active site, we also calculated plain MD trajectories in the reactant state at 10, 25, and 40 °C (300 ns at each temperature) for the two enzymes. While the backbone mobility of AHA and PPA around the catalytic residues Asp174/197 and Glu200/233 is found to be very similar (Fig. 4), it turns out that the L7 surface loop following the short helical motif carrying the active-site residues His263/299 and Asp264/300 has significantly higher flexibility in the psychrophilic enzyme, and is, in fact, the most flexible part of AHA (Supplementary Fig. 2). This effect was also seen in earlier MD simulations of the two enzymes without any bound substrate^21^, and the flexibility of this loop was indeed deemed important in earlier QM/MM simulations^19^ (where it was denoted L2).

The L7 loop (269–277/305–314) has an alanine insertion in PPA (Ala307), and its conformation becomes partly helical that apparently stabilizes its structure compared with AHA. The strictly conserved Asp264/300 upstream of the L7 loop is a critical residue that anchors the −1 position of the substrate via H bonds to the sugar 2- and 3-OH groups, and it has also been proposed to stabilize the protonated form of the general acid/base Glu200/233^37,50^. In addition, His263/299 also makes a H bond to the 3-OH group of the substrate, and is likewise strictly conserved. The effect of the increased backbone mobility of the L7 loop propagates upstream to Asp264/300 and His263/299, thereby significantly affecting also their sidechain mobilities, which are found to be higher in AHA than in PPA at all temperatures (Fig. 4). Since the global dynamics of the two enzymes is very similar, apart from the mobility of the L7 loop (Supplementary Fig. 2), it appears that the sequence change in the middle of this loop (AHA: HG−GAGNVIT vs. PPA: HGAGGSSILT) may be the main determinant of its different behavior in the two cases.

**Fig. 5:**
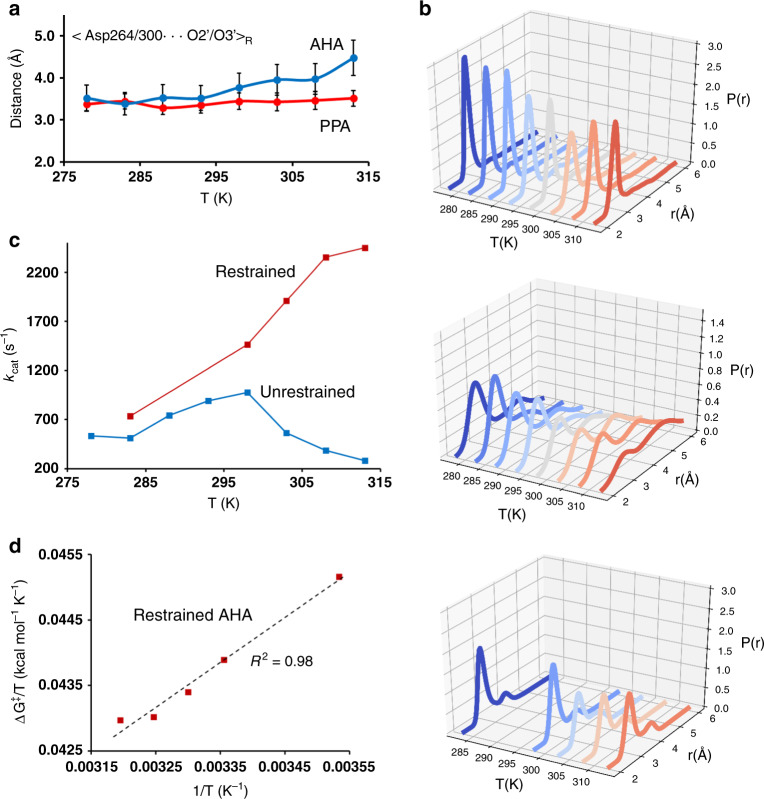
Origin of the anomalous temperature dependence of AHA. **a** Average distance between the carboxylate oxygens of Asp264/300 and the two substrate oxygens O2 and O3 of the −1 glucose unit, as a function of temperature. The curves for Asp264 in AHA and Asp300 in PPA are shown in blue and red, respectively (error bars 1 s.e.m.). **b** Probability density of the closest Asp264/300–substrate oxygen (Oδ2 – O2) distance as a function of temperature for PPA (top), AHA (middle), and the restrained AHA simulations (bottom). **c** Calculated temperature dependence of *k*_cat_ for AHA with (red) and without (blue) restraints applied to the Asp−COO^−^···O2/O3 interaction distance. **d** Arrhenius plot of Δ*G*^‡^/*T* vs. 1/*T* for the AHA simulations with distance restraints applied (s.e.m. for free energy barriers is 0.06–0.10 kcal mol^−1^).

Further analysis of the strong Asp264/300 interaction with the substrate reveals that the reactant-state probability distribution of the Asp−COO^−^···O2/O3 distances is considerably wider in AHA and also strongly temperature dependent (Fig. 5a, b). In particular, it can be seen that somewhere above room temperature, there is a notable shift in the probability distribution for AHA toward larger Asp interaction distances, which is not observed in the mesophilic enzyme. This clearly indicates that above 25 °C, where both the calculated and observed rate optima are found, the psychrophilic enzyme starts to populate a less favorable reactant state, leading to a higher free energy barrier for the reaction. In order to rigorously test this hypothesis, we repeated the EVB/MD simulations for AHA at 10, 25, 30, 35, and 40 °C with distance restraints applied to the Asp−COO^−^··· substrate interaction. As intended, these restraints change the probability distributions toward those observed in PPA (Fig. 5b), and are found to abolish the rate decay above 25 °C in the EVB/MD calculations (Fig. 5c). Hence, this computational experiment clearly suggests that it is the different temperature dependence of the substrate interaction with Asp264/300 that causes the rate optimum observed for AHA. Moreover, the Arrhenius plot for AHA with the above restraints now yields a nice linear fit (*R*^2^ = 0.98) over the entire temperature range 10–40 °C with computed values of Δ*H*^‡^ = 6.8 and *T*Δ*S*^‡^ = −6.0 kcal mol^−1^ at 15 °C (Fig. 5d). These values are thus very similar to those obtained for the unrestrained psychrophilic enzyme below its temperature optimum.

### Kinetic model for the catalytic reaction of AHA

The simplest kinetic model to describe the above situation would be that with a dead-end inhibitory state in equilibrium with the Michaelis complex leading to products

1 $$\begin{array}{ll}{\mathrm{E}} + {\mathrm{S}} \rightleftharpoons & {\mathrm{ES}}\mathop{\longrightarrow}\limits^{{k_3}}{\mathrm{E}} + P\\ & \upharpoonleft \downharpoonright {\mathrm{K}}_{{\mathrm{eq}}} \\ & {\mathrm{ES}}^{\prime} \\ \end{array}$$

**Fig. 6:**
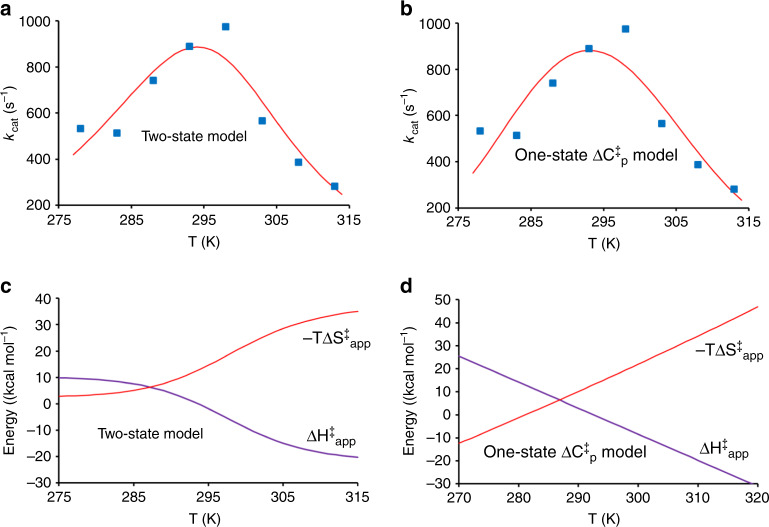
Kinetic models for the anomalous temperature dependence of AHA. **a** Fit of the two-state dead-end model to the calculated values of *k*_cat_ for the psychrophilic enzyme and (**b**) the corresponding fit of the heat capacity model to the same data points. Predictions of the two-state (**c**) and heat capacity (**d**) models of the temperature dependence of the apparent activation enthalpy and entropy contributions to the free energy barrier.

This two-state model yields $k_{\mathrm{cat}}=k_3/(1+K_{\mathrm{eq}})$ and its temperature dependence is thus determined by the thermodynamic parameters $\Delta H_3^\ddagger ,\Delta S_3^\ddagger ,\Delta H_{{\mathrm{eq}}}$, and Δ*S*_eq_. Fitting the rate curve (Fig. 3c) resulting from our EVB/MD calculations to this model yields $\Delta H_3^\ddagger = 10.2$ kcal mol^−1^, $\Delta S_3^\ddagger = - 0.00925$ kcal mol^−1^ K^−1^, Δ*H*_eq_ = 32.0 kcal mol^−1^, and Δ*S*_eq_ = +0.10746 kcal mol^−1^ K^−1^, and the corresponding curve is shown in Fig. 6a. It is clear that with only eight temperature points, the enthalpy and entropy values resulting from the fit are not very accurate but, nevertheless, give a very informative qualitative picture. That is, the kinetic model requires a strongly temperature-dependent equilibrium with the inactive state in order to capture the rate optimum. Hence, the large enthalpy cost of moving to ES’ is balanced with the entropy gain at precisely around 25 °C, where −*T*Δ*S*_eq_ = −32 kcal mol^−1^. At lower temperatures, the system will primarily reside in ES and is higher in ES’. The magnitudes of Δ*H*_eq_ and Δ*S*_eq_ appear very reasonable since they primarily would reflect the breaking of ionic H bonds between the substrate and Asp264, which are bound to be strong in terms of enthalpy. There is also always a second solution to Eq. (1) obtained by changing the sign of Δ*H*_eq_ and Δ*S*_eq_, but this can be discarded in the present case as it has a large negative activation enthalpy for the chemical step and the wrong sign of Δ*H*_eq_ and Δ*S*_eq_.

Due to the increasing population of ES’ at higher temperatures, the apparent activation enthalpy $\Delta H_{{\mathrm{app}}}^\ddagger$ will become smaller and eventually negative as it becomes dominated by −Δ*H*_eq_, the enthalpy associated with going back to ES. At that point, however, the *T*Δ*S*_eq_ penalty is larger and will dominate the apparent free energy barrier, which is what causes the rate decay in the high-temperature regime. The apparent activation enthalpy and entropy are approximately given by

2 $$\Delta H_{{\mathrm{app}}}^\ddagger \approx \Delta H_3^\ddagger - \frac{{K_{{\mathrm{eq}}}}}{{1 + K_{{\mathrm{eq}}}}}\Delta H_{{\mathrm{eq}}}$$

and

3 $$\Delta S_{{\mathrm{app}}}^\ddagger \approx \Delta S_3^\ddagger - \frac{{K_{{\mathrm{eq}}}}}{{1 + K_{{\mathrm{eq}}}}}\Delta S_{{\mathrm{eq}}}$$

where the second term in the two equations corresponds to the fractional population of ES’, for which −Δ*H*_eq_ and −Δ*S*_eq_ will add to the activation parameters of the chemical step. These expressions give the temperature-dependent apparent activation parameters shown in Fig. 6c. In the low-temperature regime around 15 °C, the model predicts a value of $\Delta H_{{\mathrm{app}}}^\ddagger$ around 6 kcal mol^−1^, in reasonable agreement with our original Arrhenius plot (Fig. 3a). It is important to note, however, that the above two-state kinetic model predicts that the apparent parameters have limiting values of $\Delta H_{{\mathrm{app}}}^\ddagger = \Delta H_3^\ddagger$ and $\Delta S_{{\mathrm{app}}}^\ddagger = \Delta S_3^\ddagger$ at low temperatures, and $\Delta H_{{\mathrm{app}}}^\ddagger = \Delta H_3^\ddagger - \Delta H_{{\mathrm{eq}}}$ and $\Delta S_{{\mathrm{app}}}^\ddagger = \Delta S_3^\ddagger - \Delta S_{{\mathrm{eq}}}$ at high temperatures.

An alternative model that has been invoked to explain the occurrence of enzyme temperature optima (that do not reflect unfolding) assumes that there is a constant difference in heat capacity between the reactant and transition states ($\Delta C_p^\ddagger \ne 0$)^30–32^. This idea is apparently inspired from studies of protein folding, where it is well-known that there is a considerable change in heat capacity between the folded and unfolded states^51^. Hence, it is hypothesized that there may also be a non-negligible heat capacity difference between the rate-limiting TS and the Michaelis complex (ES). If so, neither the activation enthalpy nor entropy will be constant, but depend on temperature according to

4 $$\Delta H^\ddagger \left( T \right) = \Delta H_0^\ddagger + \Delta C_p^\ddagger (T - T_0)$$

5 $$\Delta S^\ddagger \left( T \right) = \Delta S_0^\ddagger + \Delta C_p^\ddagger \ln \left( {\frac{T}{{T_0}}} \right)$$

where the subscript 0 denotes Δ*H*^‡^ and Δ*S*^‡^ values at a reference temperature *T*_0_. Due to the temperature dependence of Δ*H*^‡^ and Δ*S*^‡^, this model will yield curved Arrhenius plots for *k*_cat_ even for a simple one-state model, ${\mathrm{E}} + {\mathrm{S}} \rightleftharpoons {\mathrm{ES}}\mathop { \to }\limits^{k_{{\mathrm{cat}}}} {\mathrm{E}} + {\mathrm{P}}$, if $\Delta C_p^\ddagger \ne 0$. For temperature optima not related to unfolding, a negative value of $\Delta C_p^\ddagger$ would be required to fit the rate curve, and this has been interpreted such that the TS is stiffer (lower heat capacity) than the ground state^30,31^. The only experimental evidence for this type of model, however, comes precisely from curve fitting^31,32^ since the heat capacity change along an enzymatic reaction coordinate would be very difficult to measure directly.

It is again relatively straightforward to fit our calculated rate vs. temperature curve to the heat capacity model, although the fitted values of $\Delta H_0^\ddagger$, $\Delta S_0^\ddagger$, and $\Delta C_p^\ddagger$ are not very accurate since we only have eight temperature points, as noted above. However, it is an interesting qualitative exercise and the resulting fit is shown in Fig. 6b. This yields ${\Delta} H_{0}^{\ddagger}=-6.2$ kcal mol^−1^, $\Delta S_0^\ddagger$=−0.06576 kcal mol^−1^ K^−1^, and $\Delta C_p^\ddagger = - 1.13$ kcal mol^−1^ K^−1^, with *T*_0_ taken as 298 K. Comparing the above two-state dead-end model with the one-state heat capacity model (Fig. 6a, b), we see that the two theoretical curves are essentially indistinguishable. This demonstrates, beyond any doubt, that curve fitting to these kinetic models cannot be used to determine the actual mechanism behind the curved Arrhenius plot and temperature optimum. However, in our case, we now know from the analysis of conformational populations and our restrained EVB/MD experiments that it is the two-state model that is the correct one, and we have also been able to identify the nature of the ES and ES’ states. It is further interesting to note that the one-state heat capacity model predicts a very strange behavior of Δ*H*^‡^(*T*) and Δ*S*^‡^(*T*) as shown in Fig. 6d. The activation enthalpy is thus predicted to grow linearly at low temperatures toward very high values and, conversely, become large and negative at high temperatures, while the −*T*Δ*S*^‡^ term behaves in an opposite manner. This is quite different from the prediction of the two-state model where $\Delta H_{{\mathrm{app}}}^\ddagger$ and $\Delta S_{{\mathrm{app}}}^\ddagger$ reach limiting values at low and high values of *T*. Hence, the physical basis for why the activation enthalpy and entropy of an enzyme reaction would behave as predicted by the heat capacity model (Fig. 6d) is rather obscure.

## Discussion

The increasing interest in enzymes from extremophilic species and, in particular those from psychrophiles, has raised a number of fundamental questions regarding how evolution has shaped the temperature dependence of their catalytic activity and thermal stability. A major discovery in the field was the observation by Somero and coworkers^1^ that the thermodynamic activation parameters of cold-adapted enzymes have shifted toward a lower value of Δ*H*^‡^ and a more negative Δ*S*^‡^, compared with mesophilic orthologs. This finding has since then been confirmed for many different classes of enzymes^2,5^, and appears to be a universal feature of cold adaptation. The other universal feature is the reduced thermal stability that psychrophilic enzymes display^2^. The α-amylase from *P. haloplanktis* (AHA) obeys both these rules of psychrophilicity, and also shows the enigmatic behavior of becoming inactivated before protein melting sets in, as the temperature is increased^14^.

The present computer simulations reveal several hitherto hidden properties of AHA, which explain its catalytic behavior. First, it is evident from our comparison to an uncatalyzed reference reaction in solution, containing exactly the same catalytic groups as the enzyme, that a major contribution to the low activation barrier in the α-amylases originates from reduction of the reorganization free energy of the chemical reaction. This effect is often simply referred to as active-site preorganization, but can, however, be quantified, and has been seen for many other types of enzymes as well^33,47,49^. In fact, it may well be a characteristic of the glucosidases utilizing two enzyme carboxylic sidechains as their nucleophile and general acid/base for cleaving glycosidic bonds. In these reactions, there is a migration of a net negative charge over a distance of about 5 Å, which is associated with a large reorganization free energy penalty in solution. Hence, if the enzyme can provide stabilizing interaction for the negative charge at both of its carboxylate locations during the reaction, the reorganization energy can be reduced significantly. It appears that the reason why this major catalytic effect has not been revealed earlier for glucosidases, is that no comparisons to uncatalyzed water reactions have been reported in previous computational studies.

The second major result from our EVB/MD simulations at different temperatures is that, not only the free energy barriers, but also the experimental values^16^ of Δ*H*^‡^ and *T*Δ*S*^‡^ reported at 15 °C for AHA and PPA are reproduced remarkably well, with shifts of several kcal mol^−1^ in the expected direction between the two enzymes. While the 3D structures of AHA and PPA are very similar, with the same overall fold (Fig. 1a), their sequence identity is, however, only 47% with several insertions/deletions. The fact that these enzymes are also rather large, with almost 500 amino acids, thus makes it difficult to identify specific protein regions responsible for the cold adaptation of AHA. It should also be noted that the shifts in Δ*H*^‡^ and *T*Δ*S*^‡^ are smaller for the α-amylases than, e.g., endonucleases^27^, xylanases^29^, and trypsins^52^, where they are on the order of 10 kcal mol^−1^. Experimental work on AHA mutants has, however, shown that a combination of five or six AHA → PPA substitutions are sufficient for rendering mesophilic characteristics in terms of *k*_cat_, *K*_m_, Δ*H*^‡^, and *T*Δ*S*^‡^ to the psychrophilic enzyme^15^. Neither of the corresponding single mutations, on the other hand, were able to cause these changes, demonstrating that multiple contributions are at play. Interestingly, all but one (K300R) of the selected mutations were located on the protein surface, 20–25 Å away from the reaction center, which reinforces our earlier findings regarding the importance of the protein surface in cold adaptation^9–12^. However, since it is probably easier to destroy psychrophilic properties than to create them, a more critical test would be to instead identify minimal sets of mutations that confer cold adaptation to the mesophilic enzyme.

The most noteworthy result from the present computer simulations is the break in the Arrhenius plot for AHA and the corresponding calculated rate maximum at around 25 °C. This type of optimum has never been predicted by computational enzyme studies before. The optimum also occurs precisely where it is found experimentally, which is about 20 °C below the melting temperature of the enzyme^14^. We were thus able here to analyze the origin of such anomalous temperature optima in the case of AHA. Our results unambiguously show that it is primarily the Asp264 interaction with the substrate that breaks above room temperature and causes the rate decline. The evidence comes from analysis of backbone and sidechain atomic mobilities, conformational populations, and computational experiments where the interaction is maintained at higher temperatures through restraints. Hence, the kinetics of the psychrophilic enzyme is best described by a two-state model in which ES is in equilibrium with a dead-end state ES’. Such a macroscopic model yields very reasonable values for the apparent activation enthalpy and entropy as a function of temperature, and predicts that the ES ⇌ ES′ equilibrium is characterized by a large enthalpy (~30 kcal mol^−1^) that is counterbalanced by an equally large entropy term at room temperature, indicative of breaking/forming a strong interaction. The two-state dead-end model is thus similar to that proposed by Danson for explaining unusual curved Arrhenius plots in enzyme kinetics^53^. That model also adds a slow irreversible inactivation of the enzyme from the ES’ state, and describes how the rate vs. temperature curve changes with time.

We further showed here that the heat capacity model^30–32^ can fit our calculated rate curve equally well as the two-state model, although it is clearly incorrect in this case. This strongly suggests that curve fitting alone cannot be used to prove the somewhat controversial idea that $\Delta C_p^\ddagger$ could be nonzero for an elementary enzyme reaction step, and that this would be the underlying reason for curved Arrhenius plots and unusual temperature optima of the type discussed here. Moreover, we show that the two alternative models make very different predictions regarding the temperature dependence of the apparent activation enthalpies and entropies. While $\Delta H_{{\mathrm{app}}}^\ddagger$ and $\Delta S_{{\mathrm{app}}}^\ddagger$ in the two-state model reach limiting values at low and high temperatures, determined by the chemical step and inactivation equilibrium, the heat capacity model predicts a seemingly unphysical behavior of these quantities. Hence, $\Delta H_{{\mathrm{app}}}^\ddagger$ is predicted to be inversely proportional to *T* over the entire temperature range, and reaches a value of about 330 kcal mol^−1^ at 0 K for the AHA reaction. What the microscopic origin of such an enormous energy barrier would be is difficult to understand. Likewise, a large negative activation enthalpy is predicted at high temperatures for an elementary chemical reaction step, for which the physical explanation is also unclear. It should, however, be noted that the equilibrium model of Eq. (1) predicts an increased apparent heat capacity of the combined reactant state when both ES and ES’ are significantly populated, since the temperature derivative of the second term of Eq. (2) is not zero. This causes a negative dip of the apparent $\Delta C_p^\ddagger$ in the temperature region where the population transition occurs, while it goes to zero at low and high temperatures (Supplementary Fig. 3). However, in this case, the negative dip in $\Delta C_p^\ddagger$ is a consequence of the ES ⇌ ES′ equilibrium, and $\Delta C_p^\ddagger$ is not a temperature-independent constant.

Finally, it is interesting to note that, in contrast to earlier computational studies of psychrophilic enzymes, the α-amylases show distinct differences in the mobilities of the conserved active-site residues Asp264/300 and His263/299. That is, earlier work on a number of different enzymes has revealed that mutations and flexibility differences are generally located at surface loops of the protein, while the conserved active-site residues showed the same low mobility in both psychrophilic and mesophilic orthologs. Moreover, increased surface loop mobility in psychrophilic enzymes could be directly related to the changes in activation enthalpies and entropies^9–12^. The influence of protein surface residues far away from the active site on thermodynamic activation parameters is further illustrated by the multiple AHA mutations analyzed by Feller and coworkers^15^. In AHA and PPA, the catalytic groups and core of the active site do indeed show very similar and low mobility (Fig. 4), but Asp264/300 and His263/299 are clearly more flexible in the psychrophilic enzyme although the residues are totally conserved among the α-amylases^54^. The reason for this is to be found in the L7 loop downstream of the two residues, which is the most mobile part of AHA and much more flexible than in PPA. Hence, it again appears that it is mutation in a surface loop that is ultimately responsible for the different behavior of the two enzymes with regard to their temperature optima.

## Methods

### MD simulations

Models for the psychrophilic *P. haloplanktis* (AHA) and mesophilic porcine (*Sus scrofa*) pancreatic α-amylase (PPA) were based on structural information accessible in the Protein Data Bank (PDB). The highest X-ray resolution structure available for each enzyme complexed with an acarbose inhibitor was chosen, 1G94^36^ (1.74-Å resolution) for AHA and 1HX0^38^ (1.38 Å) for PPA. Acarbose was modified to represent a five-residue glucose oligomer, linked by α1–4 glycosidic bonds. Crystallographic waters were retained, except for those clashing with atoms of the modified substrate. The AHA and PPA systems were prepared for EVB/MD simulations^33,34^ by creating the enzyme–substrate complexes based on the crystallographic structures and solvating them with a 45-Å-radius spherical droplet of TIP3P water^55^ with origin at the center of mass of each complex, thereby covering the entire protein. Water molecules close to the spherical boundary were subjected to radial and polarization restraints according to the SCAAS model^56,57^. To construct a reference system for calibrating the EVB potential surface, a simplified model was used based on the conserved α-amylase active-site structure. The model comprised the sidechains of the nucleophilic group (Asp174/197) and the protonated general acid (Glu200/233), both truncated at the Cβ carbon, together with a maltose disaccharide molecule representing the substrate at the −1 and +1 positions. The reference system was solvated in a 18-Å-radius sphere of TIP3P water. The OPLS-AA/M force field^58^ was used in all calculations. Nonbonded interactions beyond a 10-Å cutoff were treated by local reaction field multipole expansion method^59^, except for the reacting groups for which all interactions were explicitly calculated. All EVB/MD calculations were carried out with the Q program^57^, using a 1-fs time step as described earlier^9^.

To calculate average backbone and sidechain-positional atomic fluctuations (RMSF), the enzyme–substrate complexes were solvated by a cubic TIP3P water box with side length 100 Å using periodic boundary conditions (PBC) in GROMACS^60^. Here, we carried out longer MD simulations of the reactant state in AHA and PPA, again using the OPLS-AA/M force field. The standard GROMACS PBC simulations involved 6 replicas of 50 ns each for both AHA and PPA at 283, 298, and 313 K and used a 2-fs time step.

### DFT cluster model of reference reaction

The DFT cluster model was used to determine the structure and energetics of the rate-limiting glycosylation transition state in a continuum solvent model. The model consisted of the same chemical groups as those used in the EVB reference system, except that the amino acid sidechains were truncated at the Cα carbon. The glucose ring at the −1 position was proposed to be in half-chair conformation in the initial TS structure guess (see Fig. 2c for the resulting optimized geometry). The energetics of this cluster model was evaluated with the M06-2X functional^39^ using the Gaussian09 program^61^. The transition state was first optimized, and then geometry optimization was carried out toward the reactant and intermediate states, using the 6–31 G(d,p) basis set. The α-carbons of the two amino acid sidechains were kept fixed during the optimizations. Electronic energies were then recalculated for the optimized geometries with the larger 6–311+G(2d,2p) basis set, with solvent effects from surrounding water estimated using SMD model^40^. Zero-point energies were obtained from frequencies calculated at the same level of theory as geometry optimization. Free energy estimates were thus obtained by adding the zero-point energy and solvent contributions to the electronic energies obtained by the large basis set.

### EVB/MD calculations of free energy profiles

The systems were first equilibrated for 246 ps at increasing temperatures, starting from 1 K, and heated in a stepwise manner toward the final desired temperature for EVB/MD production runs. EVB/MD free energy perturbation simulations^11,34^ were carried out with 51 discrete *λ* windows, starting at *λ*_1_ *=* *λ*_2_ = 0.5, near the transition state, and propagated toward reactants (*λ*_1_ = 1) and intermediate (*λ*_2_ = 1). To calibrate the EVB reaction potential energy surface, the average free energy profile from 250 independent simulations for the reference reaction system in water was fitted to the corresponding free energy profile obtained by the DFT cluster calculations at 298 K. The temperature dependence of the reference reaction was analyzed, after calibration, by repeating the free energy profile calculations also at 283, 290.5, 305.5, and 313 K, again using 250 replicas at each temperature. Each individual free energy profile encompassed 510 ps, and the total simulation time for obtaining the reference system data was thus 0.64 μs.

The enzyme EVB/MD simulations for AHA and PPA were run at eight different temperatures from 278 to 313 K, with 5-K intervals. At each temperature, about 300 randomized individual replicate simulations were carried out to obtain the free energy profiles, using the same free energy perturbation scheme^11^ as above. In addition, a system with added distance restraints was analyzed for AHA. Weak harmonic restraints between the His263 and Asp264 sidechains of the enzyme and the O2 and O3 hydroxyl groups of the substrate glucose in the –1 position were then imposed during the EVB/MD calculations. This restrained system was simulated at 283, 298, 303, 308, and 313 K, again with 300 randomized replicas per temperature. The total simulation time of all enzyme EVB/MD calculations amounted to 3.2 μs. Fitting of enzyme activity curves to kinetic models was done with Gnuplot (http://www.gnuplot.info).

### Reporting summary

Further information on research design is available in the Nature Research Reporting Summary linked to this article.

## Supplementary information

Supplementary Information

Reporting Summary

Description of Additional Supplementary Files

Supplementary Data 1

## Data Availability

The data that support this study are available from the authors upon reasonable request.

## Code availability

Source code for the Q program^57^ is available at https://github.com/qusers/Q6, or upon request from J.Å.
