# Supplementary material for: Computer simulations explain the anomalous temperature optimum in a cold-adapted enzyme
Source: Nat Commun. 2020 May 26;11:2644. doi: 10.1038/s41467-020-16341-2 (PMC7250929; doi:10.1038/s41467-020-16341-2)
Supplement: Supplementary file 1 — Supplementary Information [file 41467_2020_16341_MOESM1_ESM.pdf]

Supplementary Information

Computer simulations explain the anomalous temperature optimum in a cold-adapted enzyme

Sočan *et al*.

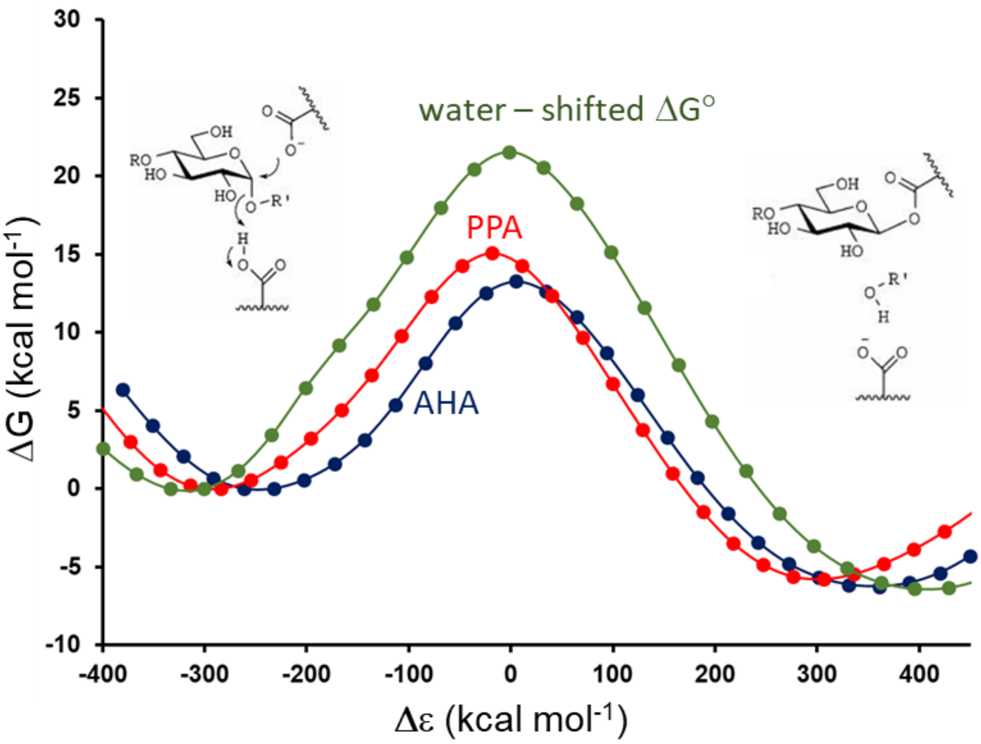

Supplementary Figure 1. Stabilization of the covalent intermediate does not suffice to explain the catalytic effect in the two enzymes. Calculated reaction free energy profiles as in Fig. 2, except that the reaction free energy (ΔG°) for uncatalyzed water profile has been shifted (by reparametrization of the EVB potential surface) to coincide with the calculated value in AHA. It is evident that the barrier for the water reaction is still about 8 kcal/mol higher than in AHA, which is due to a larger reorganization free energy in the water reaction.

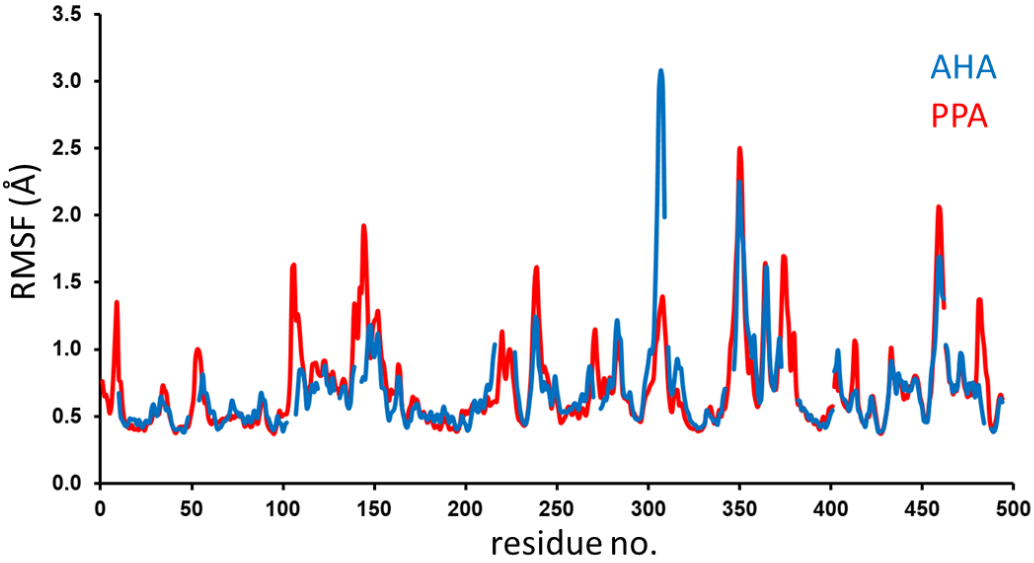

Supplementary Figure 2. Overall backbone mobility of AHA and PPA. Calculated average backbone positional root-mean-square fluctuations per residue in the reactant state for AHA (blue) and PPA (red) along the amino acid sequence numbered according to PPA.

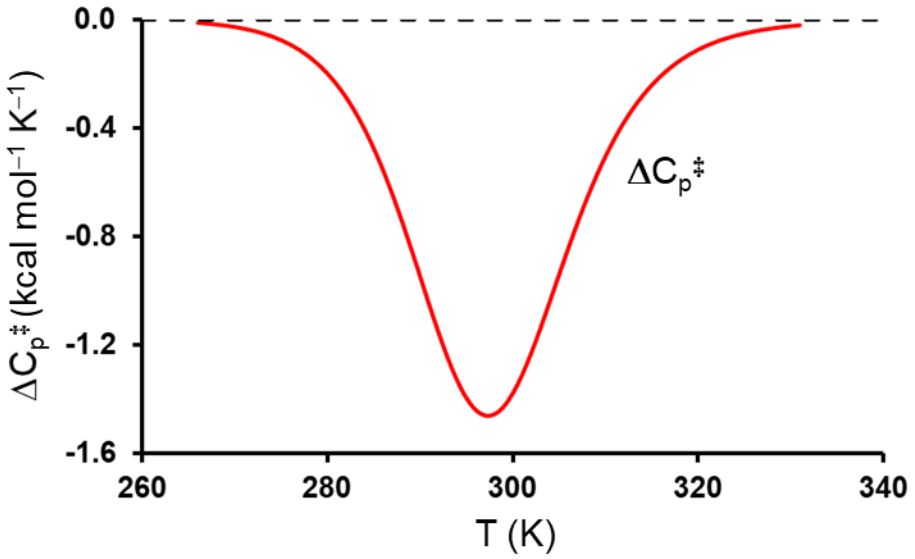

Supplementary Figure 3. Heat capacity behaviour of the equilibrium model. Calculated activation heat capacity difference from Equation (2), $\Delta C_p^{\ddagger} = \partial \Delta H_{app}^{\ddagger} / \partial T$, where the fitted values of $\Delta H_3^{\ddagger}$, ΔH_{eq} and ΔS_{eq} are used.
