# Supplementary material for: Computer simulations explain the anomalous temperature optimum in a cold-adapted enzyme
Source: Nat Commun. 2020 May 26;11:2644. doi: 10.1038/s41467-020-16341-2 (PMC7250929; doi:10.1038/s41467-020-16341-2)
Supplement: Supplementary file 3 — Description of Additional Supplementary Files [file 41467_2020_16341_MOESM3_ESM.pdf]

Description of Additional Supplementary Files

File name: Supplementary Data 1

Description: Our former Supplementary Table 1 containing coordinates and energies of optimized structures.
